# Chemosynthetic ectosymbionts associated with a shallow-water marine nematode

**DOI:** 10.1038/s41598-019-43517-8

**Published:** 2019-05-07

**Authors:** Laure Bellec, Marie-Anne Cambon Bonavita, Stéphane Hourdez, Mohamed Jebbar, Aurélie Tasiemski, Lucile Durand, Nicolas Gayet, Daniela Zeppilli

**Affiliations:** 1 IFREMER, Centre Brest, REM/EEP/LEP, ZI de la pointe du diable, CS10070, 29280 Plouzané, France; 2 IFREMER, Univ Brest, CNRS, Laboratoire de Microbiologie des Environnements Extrêmes, F-29280 Plouzané, France; 3 CNRS, UMR 6197-Laboratoire de Microbiologie des Environnements Extrêmes (LM2E), Institut Universitaire Européen de la Mer (IUEM), Technopole Brest-Iroise, Plouzané, France; 4 Université Bretagne Occidentale (UBO), UMR 6197 - Laboratoire de Microbiologie des Environnements Extrêmes (LM2E), Institut Universitaire Européen de la Mer (IUEM), Technopole Brest-Iroise, Plouzané, France; 5 Station biologique de Roscoff, UMR 7144 CNRS-SU, Adaptation and Biology of Invertebrates in Extreme Environment team, Place G. Teissier, 29680 Roscoff, France; 6 Present Address: Observatoire Oceanologique de Banyuls-sur-Mer, UMR 8222 CNRS-SU, 1 avenue Pierre Fabre, 66650 Banyuls-sur-Mer, France; 7 Université Lille, CNRS, UMR 8198 - Evo-Eco-Paleo, SPICI group, 59000 Lille, France

**Keywords:** Biodiversity, Environmental microbiology

## Abstract

Prokaryotes and free-living nematodes are both very abundant and co-occur in marine environments, but little is known about their possible association. Our objective was to characterize the microbiome of a neglected but ecologically important group of free-living benthic nematodes of the Oncholaimidae family. We used a multi-approach study based on microscopic observations (Scanning Electron Microscopy and Fluorescence *In Situ* Hybridization) coupled with an assessment of molecular diversity using metabarcoding based on the 16S rRNA gene. All investigated free-living marine nematode specimens harboured distinct microbial communities (from the surrounding water and sediment and through the seasons) with ectosymbiosis seemed more abundant during summer. Microscopic observations distinguished two main morphotypes of bacteria (rod-shaped and filamentous) on the cuticle of these nematodes, which seemed to be affiliated to *Campylobacterota* and *Gammaproteobacteria*, respectively. Both ectosymbionts belonged to clades of bacteria usually associated with invertebrates from deep-sea hydrothermal vents. The presence of the *AprA* gene involved in sulfur metabolism suggested a potential for chemosynthesis in the nematode microbial community. The discovery of potential symbiotic associations of a shallow-water organism with taxa usually associated with deep-sea hydrothermal vents, is new for Nematoda, opening new avenues for the study of ecology and bacterial relationships with meiofauna.

## Introduction

Symbioses between animals and chemosynthetic bacteria are widespread across diverse ecosystems, ranging from shallow-water coastal sediments to the deep-sea^1^. Our knowledge to date indicates that chemosynthetic symbioses can be found in diverse coastal sediments, as well as in limestone caves (freshwater amphipod host^2^) or in the low sulfide sediments of Elba Island in the Mediterranean (oligochaete worms^3^). Hundreds of species from different phyla are known to harbour chemosynthetic symbioses, especially for hydrothermal vent organisms such as shrimps, crabs, gastropods, polychaetes and mussels^1,4^. So far, most of the known symbioses involve macrofaunal hosts. However, meiofaunal organisms, small benthic invertebrates such as nematodes and copepods living in aquatic systems, could also potentially be important hosts of chemosynthetic symbioses. On-going work on small organisms and microbiome characterisation will likely reveal many more chemosynthetic symbioses, such as that reported in a recent study from a deep-sea hydrothermal vent nematode on the Mid-Atlantic Ridge^5^.

Shallow-water nematode-bacteria associations have been described in only two sub-families of nematodes: Stilbonematinae and Astomonematinae. Both sub-families are associated with reduced conditions such as the subsurface intertidal layers of sulfur-rich sediments^6^ or sublittoral methane sources^7^. Despite a different feeding ecology, these two sub-families of nematodes harbour bacterial symbionts displaying high sequence similarity^8^. To date, studies of nematode-associated microbiomes are still rare, especially in marine habitats. The native microbiome of the model species *Caenorhabditis elegans* analysed from wild specimens of different substrates showed a species-rich bacterial community distinct from its habitat and congeneric species. Furthermore, experiments indicated that bacteria could improve host fitness under stressful conditions and participate in defence against pathogens^9^. Among plant-parasitic nematodes, significant effects of the soil type and nematode species on microbiomes were revealed^10^. In contrast, the microbiome of *Pristionchus pacificus*, a necromenic nematode associated with scarab beetles, has a remarkably stable microbiome^11^. In marine nematodes, a comparison of bacterial communities of three cryptic species of *Litoditis marina* demonstrated the existence of species-specific microbiomes. A controlled feeding experiment revealed evidence that despite morphological similarity and a shared feeding habit (bacterivory), these cryptic species have differences in their associated microbiomes^12^. A recent study explored patterns in numerous marine nematode microbiomes from three distinct geographic areas and multiple habitats^13^. Surprisingly, microbial communities have shown no geographical patterns (oceanic region or habitat type) and no correlation with host phylogeny or feeding morphology. These few studies with contrasting results demonstrate the need for further nematode-microbial association studies.

In the present study, we aimed to improve the current knowledge on shallow-water meiofauna microbiomes, a neglected but ecologically important group in the benthic system. Does the free-living marine nematode associated microbial community differ from their surrounding environment, that is sediment and water samples? Does the microbial community have a metabolic role? We focused on a free-living nematode from the Oncholaimidae family, *Metoncholaimus albidus* (Bastian, 1865^14^), regularly isolated from Roscoff (Brittany coast, France). We report observations on the morphology (measurements and Scanning Electron Microscopy (SEM)) and genetics (nuclear and mitochondrial markers) of this nematode. The bacterial community characterization is described based on microscopic observations (SEM and Fluorescence *In Situ* Hybridization (FISH)), coupled with molecular diversity based on the 16S rRNA metabarcoding. We also investigated a potential metabolic role of bacteria using a gene involved in sulfur metabolism.

## Results

### Morphology and molecular identity of the free-living marine nematode: *M*. *albidus*

We identified the most abundant nematode recovered in Roscoff as *M*. *albidus*. We used multiple morphological measurements for males, females and juveniles (for details see Supplementary file 1) and SEM micrographs (Supplementary file 2) for identification. Additionnaly, we performed phylogenetic analyse suggesting that sequences of the free-living marine nematode from the old harbour of Roscoff formed a clade with maximum support thus representing a single species (Supplementary file 3).

### Cloning versus metabarcoding

Bacterial composition of three *M*. *albidus* (Ma_Rm03, Ma_Rm32 and Ma_Rm34) was assessed concurrently with two methods (cloning and metabarcoding) on the same DNA extracts. Results showed that sequences produced by cloning or metabarcoding were related to the same lineages for the three *M*. *albidus*, however with differences on their relative abundance (for details see Supplementary file 4). In light of this evidence, we used metabarcoding for all samples (nematodes, sediment and water).

### Negative control of metabarcoding

Three analyses of negative controls were performed under FROGS: one with nematodes and a DNeasy Blood & Tissue blank, one with sediment and a DNeasy PowerMax Soil blank, and one with water and a DNeasy PowerWater blank. After the bioinformatics procedures, a selection of OTUs (>1% of total reads) was compared for each dataset. Overall, the analyses showed that there were no OTUs in common between blanks and nematodes, blanks and sediment, and blanks and water (Supplementary file 5).

### Bacterial communities

The metabarcoding approach (region V3-V4 of the 16S rRNA gene) used to characterize bacterial communities associated with 25 *M*. *albidus*, three sediment samples, and three water samples produced a total of 4,449434 reads after filtration and taxonomic assignment (Supplementary file 6). The reads were clustered in 801 OTUs taxonomically assigned with the Silva 128 database (Supplementary file 7). The bacterial community of 25 *M*. *albidus* from Roscoff was dominated by *Proteobacteria*-related sequences, which represented 47% of all sequences, followed by *Fusobacteria* (11%) and *Bacteroidetes* (10%). Two datasets were created and analysed separately: one composed of OTUs from the three environments (nematode, sediment and water) sampled on the same day (July 2017) and one dataset included OTUs of 25 *M*. *albidus* from four seasons (autumn 2016, winter 2017, spring 2017 and summer 2017).

### Bacterial communities from three environments in summer

**Figure 1:**
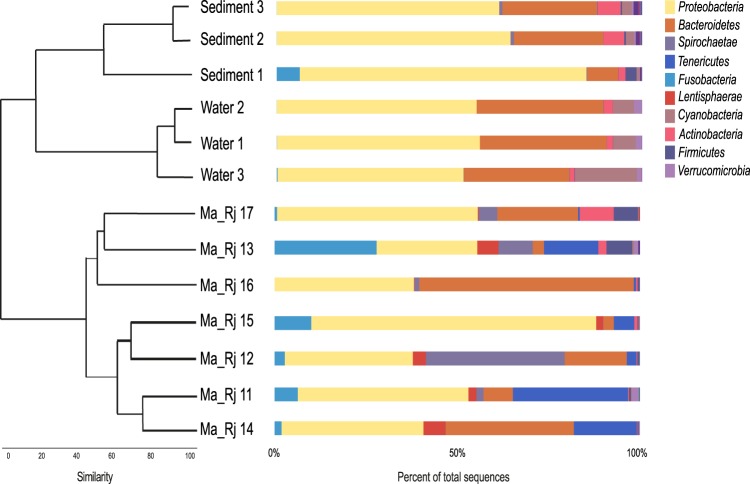
Cluster diagram based on bacterial community similarity for three environments in summer at Roscoff. Left: Bray-Curtis index showing the similarity among the bacterial community of nematode, water and sediment samples collected in July 2017. Right: relative abundance of bacterial community from nematode, water and sediment samples and their taxonomic assignment.

In July 2017, we compared the microbial diversity of seven *M*. *albidus*, three sediment samples and three water samples. The overall bacterial community composition was notably different among the three environments (Fig. 1). The bacterial community composition is characterized as it follows: for nematode, *Proteobacteria* with 46% (mostly *Gammaproteobacteria* and *Deltaproteobacteria*), *Bacteroidetes* with 21% and *Tenericutes* with 10% dominated; for sediment samples, *Proteobacteria* with 68% (mostly *Gammaproteobacteria*, *Campylobacterota*, and *Deltaproteobacteria*), by *Bacteroidetes* with 20% and *Actinobacteria* with 5% dominated; for water samples, *Proteobacteria* with 54% (mostly *Alphaproteobacteria* and *Gammaproteobacteria*,), by *Bacteroidetes* with 33% and *Cyanobacteria* with 10% dominated. Nematode bacterial diversity seemed to be variable, especially for Ma_Rj12 and Ma_Rj16. Sediment and water samples were uniform in their bacterial diversity and abundance.

Alpha diversity index values for each environment are shown in Table 1. We observed that for nematodes bacterial communities, richness (number of observed OTUs) and Chao1 (richness + estimated number of unobserved OTUs) were close, suggesting that almost all OTUs were detected. These observations were confirmed by the slopes of rarefaction curves (Supplementary file 8A). Water and sediment samples had greater diversity than *M*. *albidus*, with >331 OTUs. A high value for the Shannon index indicates high evenness in a sample, as in Ma_Rj13 and Ma_Rj17 (many OTUs with few sequences, i.e. low dominance) whereas a low value shows a high dominance of a few OTUs in the bacterial community. For example, the bacterial community of Ma_Rj15 was dominated by only two OTUs: *Gammaproteobacteria*, *Thiothrix* (54%) and *Deltaproteobacteria*, *Desulfobulbus* (20%).

**Table 1:** Alpha diversity indices. OTUs numbers, species richness (Chao1 and standard error), Shannon and InvSimpson indices.

Sample ID	OTU	Chao1 ± SE	Shannon	InvSimpson
Water1	383	440 ± 17	3.52	15.70
Water2	331	420 ± 24	3.45	16.11
Water3	444	504 ± 19	3.97	24.19
Ma_Rj11	135	196 ± 23	2.15	5.18
Ma_Rj12	142	203 ± 27	1.86	4.06
Ma_Rj13	141	204 ± 29	2.93	8.21
Ma_Rj14	166	217 ± 20	2.05	4.70
Ma_Rj15	130	163 ± 15	1.63	2.92
Ma_Rj16	116	184 ± 29	2.10	5.84
Ma_Rj17	312	357 ± 15	3.79	20.59
Sediment1	408	486 ± 25	3.81	18.23
Sediment2	426	478 ± 17	4.11	12.08
Sediment3	395	419 ± 10	4.24	15.14

Community structure analyses of environments were performed with Beta diversity indices, thus making it possible to understand relationships between bacterial communities. A cluster diagram of similarity (Fig. 1) showed a clear separation between the different environments. Water and sediment samples clustered at 20% whereas *M*. *albidus* samples were separated into two groups: one with Ma_Rj13, 16 and 17 and the second with Ma_Rj15, 12, 11 and 14. A Venn diagram with significant OTUs (number of sequences >0.1% of total sequences) revealed that only one OTU was shared among the three environments; similarly, only one OTU was common between nematode and sediment (Supplementary file 8B). The MANOVA analysis, based on the beta diversity distance matrix detected significant differences among the different environments, suggesting that the factor ‘environment’ could explain 55% of the total bacterial variation in summer.

**Figure 2:**
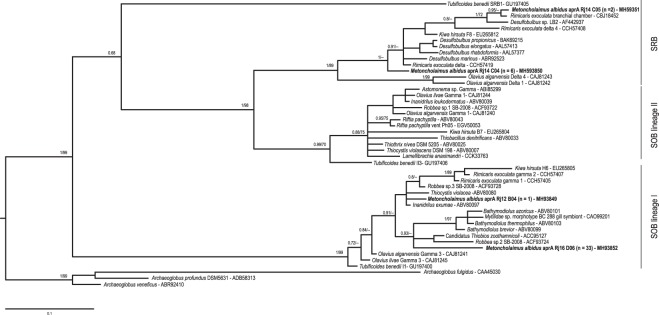
Phylogenetic tree of *AprA* gene by BI and ML. The numbers are posterior probabilities (BI) and bootstrap proportions (ML) reflecting clade support (values below 75 are indicated by dashes). Sequences representing specimens from this study are shown in bold where n represents the number of clones. Three *Archaeoglobus* sequences were used as the outgroup.

A phylogenetic analysis was performed with four *AprA* representative sequences of bacteria from *M*. *albidus* collected during summer (Fig. 2). For simplicity, only the BI tree is shown; the ML tree has the same topology. The majority of *AprA* sequences obtained (80%) belonged to lineage I sulfur-oxidizing bacteria (SOB) and the rest were related to sulfate-reducing bacteria (SRB). All sequences, except one (*M*. *albidus* aprA Rj12 B04) belonged to a clade comprising sequences from mussel symbionts and one from nematode symbiont. No sequence of *M*. *albidus* grouped with those in the SOB lineage II contrary to many nematodes or worms, as gutless oligochaete worm and giant worm, *AprA* sequences. One representative sequence (*M*. *albidus* aprA Rj14 C05) belonging to SRB was close to *Rimicaris exoculata* symbiont sequences.

### Temporal variation of bacterial communities associated with *M*. *albidus*

**Figure 3:**
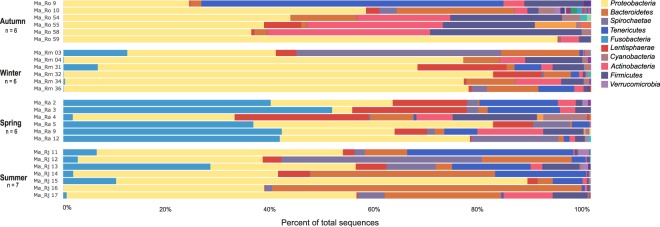
Bacterial community distribution of 25 *M*. *albidus* at the phylum level. The relative abundance is represented in terms of percentage of total effective bacterial sequences per sample.

We observed variability in the relative abundance and microbial composition among seasons (Fig. 3). In autumn, the bacterial community was dominated by *Proteobacteria*-related sequences, which represented 49% of total reads, followed by *Actinobacteria* (14%) and *Firmicutes* (14%). Among the *Proteobacteria*, reads were related to *Gammaproteobacteria* (59%) and *Betaproteobacteria* (23%). In winter, the bacterial community was dominated by *Proteobacteria*-related sequences, which represented 66% of total reads, followed by *Bacteroidetes* (8%) and *Spirochaetae* (7%). Among the *Proteobacteria*, reads were related to *Gammaproteobacteria* (68%) and *Campylobacterota* (27%). In spring, the bacterial community was dominated by *Fusobacteria*-related sequences, which represented 35% of total reads, followed by *Proteobacteria* (27%) and *Lentisphaerae* (13%). Among the *Proteobacteria*, reads were related to *Gammaproteobacteria* (74%) and *Alphaproteobacteria* (10%). In summer, the bacterial community was dominated by *Proteobacteria*-related sequences, which represented 46% of total reads, followed by *Bacteroidetes* (21%) and *Tenericutes* (10%). Among the *Proteobacteria*, reads were related to *Gammaproteobacteria* (73%) and *Deltaproteobacteria* (17%).

Within each season, overall similarities across individuals were observed. There were also cases where the bacterial diversity was clearly dominated by few OTUs. For example, abundances of nematodes Ma_Ro59 and Ma_Ra5 were associated to one OTU related to *Gammaproteobacteria* (85 and 40% respectively) whereas Ma_Rj16 was dominated by two OTUs of *Bacteroidetes* (50%).

Overall, *M*. *albidus* bacterial community was dominated by sequences representing *Gammaproteobacteria*, however clear differences at lower taxonomic rank (i.e. genus level) were observed (Supplementary file 9). For example, *Gammaproteobacteria*-related sequences were mostly affiliated to *Thiothrix* and to *Pseudoalteromonas* in summer and winter, respectively. *Campylobacterota* were mainly present in winter and highly dominated by *Arcobacter*, while *Deltaproteobacteria* were mainly observed in summer and dominated by *Desulfobulbus* (Supplementary file 9). Among the *Campylobacterota*, the analysis revealed 31 OTUs representing five genera (*Arcobacter*, *Campylobacter*, *Sulfurospirillum*, *Sulfurimonas* and *Sulfurovum*). Sequences related to *Sulfurovum* were separated into five OTUs. BLAST searches showed a high similarity with an *Campylobacterota* ectosymbiont of *Tubificoides benedii* (98–100%) and a cultured bacterium *Sulfurovum lithotrophicum* (97–99%).

**Figure 4:**
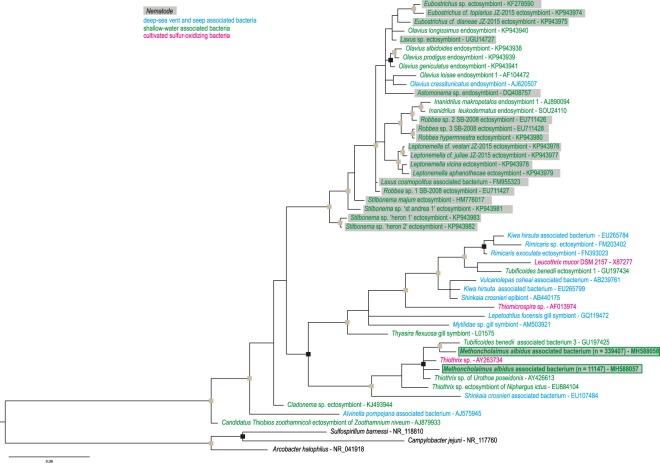
Bayesian inference tree based on the 16S rRNA gene for *Gammaproteobacteria*. Representative sequence names in squares are from this study where n is the number of sequences. At nodes, grey squares correspond to posterior probabilities >0.95; black squares correspond to posterior probabilities >0.9. Three *Campylobacterota* sequences were used as the outgroup.

Among the *Gammaproteobacteria*, the cluster analysis revealed 190 OTUs representing many genera. In particular, two OTUs observed in the summer represented 27% and 75%, respectively of the total summer *Gammaproteobacteria* relative abundance. A phylogenetic analysis was performed with these two sequences and addidional *Gammaproteobacteria* sequences from deep-sea and shallow-water habitats (for simplicity, only the BI is shown) (Fig. 4). Both sequences of *M*. *albidus* grouped with sequences representing the genus *Thiotrix*. The clade was formed by a cultivated sulfur-oxidizing *Thiothrix*, two *Thiothrix* associated with amphipods and one bacterium associated with a marine oligochaete worm. A sister group was mainly composed of sequences from bacteria associated with deep-sea hydrothermal vent organisms such as shrimp (*Rimicaris*), mussel (*Mytilidae*), crab (*Kiwa*), squat lobster (*Shinkaia*), barnacle (*Vulcanolepas*) and snail (*Lepetodrilus*). All endo- or ectosymbionts from marine nematodes (*Astomonema*, *Eubostrichus*, *Laxus*, *Leptonemella*, *Robbea*, and *Stilbonema*) available in GenBank formed a large group with additional shallow-water organisms.

### Observations of bacteria using SEM analyses

**Figure 5:**
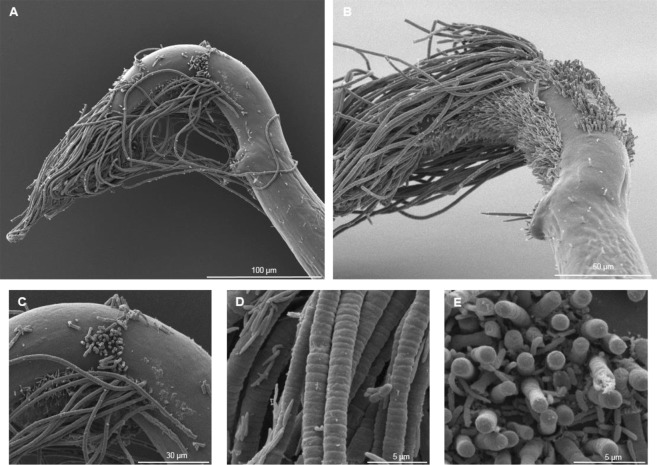
SEM micrographs of bacteria from *M*. *albidus*. (**A**) Diversity of bacteria on the posterior region, (**B**) posterior region of a male with filamentous bacteria at different stages, (**C**) filamentous bacteria directly attached to the cuticle, (**D**) close-up showing small subunits of filamentous bacteria, (**E**) bacterial community (filamentous and rod-shaped).

SEM observations of 18 specimens revealed the presence of bacteria with distinct morphologies (filamentous and rod-shaped) on the cuticle. Filamentous bacteria were observed on nematodes sampled in July 2016 and July 2017 but not in November 2016. Bacteria could be found on the entire body but most bacterial coverages were observed at the posterior part (Fig. 5A,B). The main bacterial morphotype was very long filaments, directly attached on the cuticle (Fig. 5B,C) and seemed to be composed of small subunits (Fig. 5D). Other bacteria less represented were observed on and between the filamentous ones, they were smaller and rod-shaped (Fig. 5D,E). This type of bacteria was also observed in large numbers on the posterior girdle of some female specimens (Supplementary file 2C–F). None of the nematodes examined harboured bacteria in their mouth cavities.

### Observations of bacteria using FISH analyses

**Figure 6:**
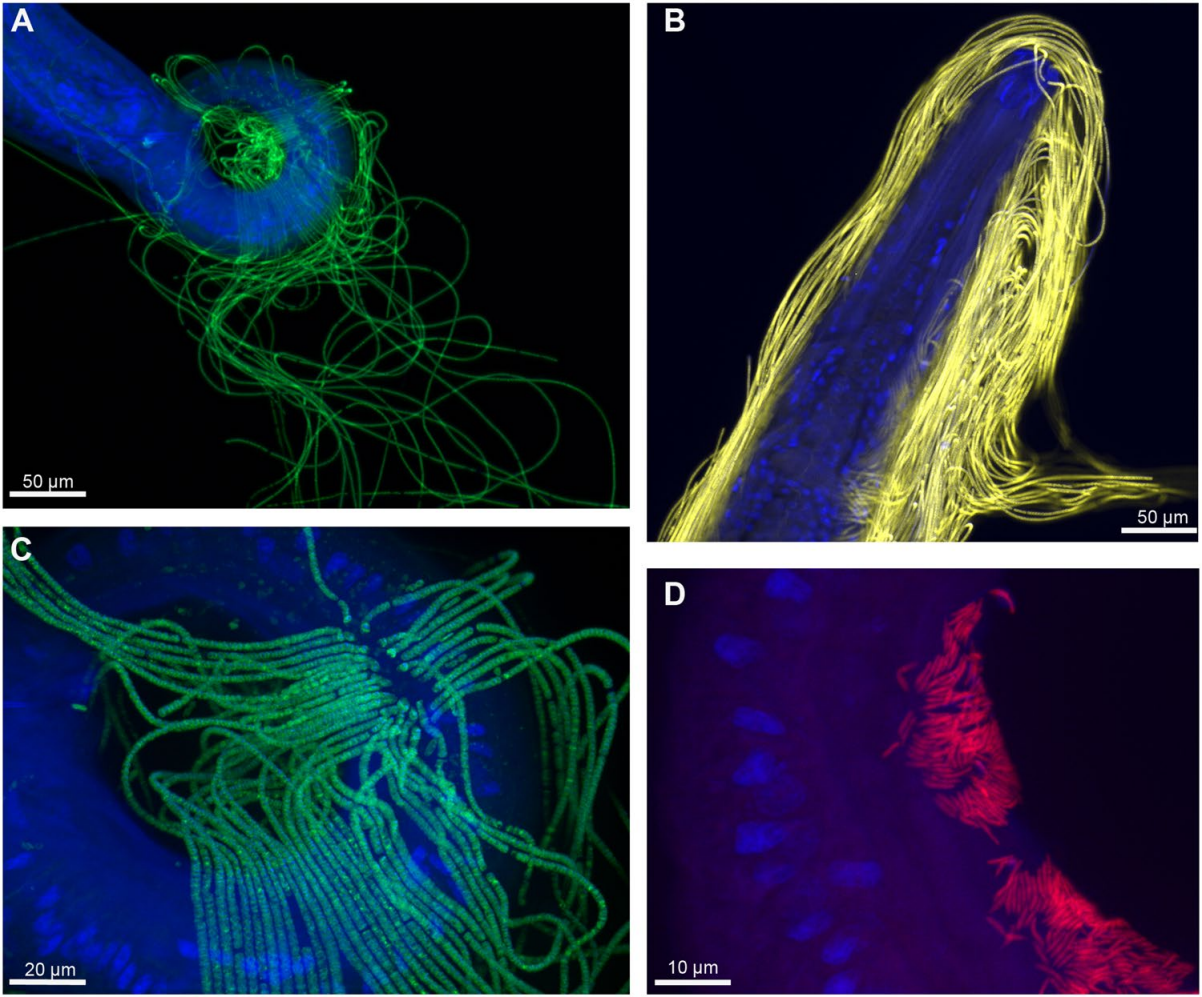
FISH of bacteria from *M*. *albidus* (July 2017). In blue, DAPI-stained host nuclei; in yellow, bacteria hybridized with the general probe targeting Eubacteria; in red, bacteria hybridized with the specific probe targeting *Campylobacterota*; in green, bacteria hybridized with the specific probe targeting *Gammaproteobacteria*. (**A**) posterior region of a male colonized by filamentous *Gammaproteobacteria*, (**B**) anterior region of a *M*. *albidus* colonized by filamentous *Eubacteria*, (**C**) close-up of small subunits and attachment of filamentous *Gammaproteobacteria* on the cuticle, (**D**) mat of rod-shaped *Campylobacterota* colonizing the cuticle of the host.

Thirteen *M*. *albidus* were observed with FISH to reveal the occurrence of bacteria. FISH observations with a general bacterial probe revealed the presence of a main bacterial morphotype (long filamentous bacteria) together with a less abundant morphotype with small rods on the cuticle, similar to those observed by SEM (Fig. 6). Nematodes sampled in July 2016 and July 2017 harboured a large number of filamentous bacteria on the tail (Fig. 6A) and anterior region (Fig. 6B). For a deeper understanding of the bacterial species composition, specific probes were used to detect specific microbial taxa, alone and in co-hybridization with the general eubacterial probe (Supplementary file 10). Positive results were observed with a *Gammaproteobacteria* probe for the filamentous bacteria and with a *Campylobacterota* for the rod-shaped mat (Fig. 6C,D). We found no fluorescence with the *Deltaproteobacteria* probe indicating low number of cells or dormant ones. We also checked for autofluorescence and the absence of signal using non-hybridized specimens and nonsense probe (negative controls).

## Discussion

### *Metoncholaimus albidus*-associated bacteria

In this study, we show that *M*. *albidus* harbours a bacterial community distinct from those of the water and the sediment from which the nematodes were collected. The bacterial community varies among all seasons and epicuticular bacteria are only obvious in the summer samples. Microscopic observations (SEM and FISH) allowed us to distinguish two main morphotypes of bacteria (small mat of rod-shaped and a majority of long filamentous) on the cuticle that seem to be affiliated to *Campylobacterota* and *Gammaproteobacteria*, respectively. Additionaly, metabarcoding results showed that the symbionts of *M*. *albidus* were mainly affiliated to *Thiotrix* (*Gammaproteobacteria)*, to *Desulfobulbus* (*Deltaproteobacteria*, but not detected in FISH), and to *Thiovulgaceae* (*Campylobacterota*) in summer. The genus *Thiothrix* is composed by long filamentous bacteria^15^ whereas *Desulfobulbus* is ellipsoidal to lemon-shaped bacteria^16^.

Free-living marine nematodes are an important component of anoxic and sulfidic benthic habitats^17^, where they often seem to establish symbioses with sulfur bacteria. In marine organisms, three main forms of symbioses are recognized: (i) ectosymbiotic associations (symbionts attached to the outside of the host), and two types of endosymbiotic associations: (ii) symbionts inside the host extracellularly or (iii) intracellularly. Endosymbiotic bacteria have previously been described in the nematode genera *Astomonema* and *Parastomonema*^6,18^. Astomonematines are mouthless and lack an oesophagus, therefore depending entirely on their bacterial symbionts for their nutrition. They are associated with reduced conditions such as oxygen-poor sediments^6^. Chemoautotrophic ectosymbionts have so far been described in at least eight genera and numerous species of the subfamily Stilbonematinae^19^. The Stilbonematines are long in form, widespread around the world and particularly abundant in carbonate sands of tropical and subtropical shallow-water habitats. Symbionts are located in the thin layer of mucus produced by glandular sensory organs that are unique to the Stilbonematines^20^. The bacteria differed in size, morphology, arrangement, and were described as chemolithotrophs.

To date, in marine nematodes, microbial studies have focused on the discovery and characterisation of the few ‘visually obvious’ symbionts, but rarely on the whole ‘microbiome’. Schuelke *et al*.^13^ showed that for 33 distinct morphological genera, no clear pattern in host-associated symbionts could be identified according to host morphology, ocean region or feeding ecology. As for *M*. *albidus*, the microbiomes associated with the worms were clearly distinct from the microbial assemblages in the surrounding sediments. Contrary to Schuelke *et al*.^13^, our study focuses on a single nematode species (and its surrounding habitat, sediment and water). In shallow water, Debrycke *et al*.^12^ focused on three cryptic species of the bacterivorous marine nematode *Litoditis marina* and showed that there were species-specific microbiomes with a high interindividual variability suggesting niche partitioning and different feeding strategies. Our study of *M*. *albidus* (one host and one location), shows high seasonal variability that could be mainly due to the *Gammaproteobacteria* epibiont population, and without high interindividual variability. Further studies of microbiomes of free-living marine nematodes from a large range of genera and geographical locations in shallow-water areas are essential for a better and deeper understanding of feeding ecology and biogeographic relationships. The association of *M*. *albidus* with two main types of bacteria in summer is reminiscent of Stilbonematine nematodes and could involve a recognition mechanism allowing some bacteria to attach and grow on the surface of the worms while others cannot^19^. In some of the species studied, this mechanism involves a C-type lectin that, when perturbed, triggers the detachment of the epibiotic bacteria^21,22^.

### Bacterial seasonal variability

FISH observations (done in July) and metabarcoding results showed that the ectosymbionts of *M*. *albidus* were mainly affiliated to *Gammaproteobacteria*, *Deltaproteobacteria*, but not detected in FISH, and to *Campylobacterota* in summer. In winter, *Gammaproteobacteria* were mainly related to *Pseudoalteromonas*, *Deltaproteobacteria* were almost absent, and *Campylobacterota* were much higher according to metabarcoding approach. Our results show a significant seasonal variation of symbionts associated with this shallow-water nematode, suggesting a metabolic shift from heterotrophic bacteria to autotrophic bacteria between seasons. Indeed, bacteria affiliated to the genus *Desulfobulbus* inhabit anaerobic freshwater mud as well as marine sediments and are known as important sulfur-reducers^16,23^. The genus *Thiothrix* is composed of filamentous sulfur-oxidizing bacteria that form gliding gonidia and rosettes in various habitats^15^. *Thiothrix* spp. have been initially described from sulfide-containing natural waters and irrigation systems^24–26^, but were also found in a terrestrial ecosystem, the sulfide-rich Frassassi limestone cave^2^. A comparative sequence analysis of the 16S rRNA gene has revealed that sequences of *M*. *albidus* were close to a marine phylotype of *Thiothrix* that lives on a small free-living amphipod crustacean, *Urothoe poseidonis*^27^ and a *Gammaproteobacteria* of a marine oligochaete worm *Tubificoides benedii*^28^. Filamentous *Gammaproteobacteria* embedded in the cuticle of the posterior region of the oligochaete worm were discovered first^29^ and genetically characterized later^28^ as *Thiothrix* spp. Morphological similarity to sulfur-oxidizing bacteria, a strong hydrogen sulfide smell at the collection site, high specific association^29^ and genetic characterisation close to the *Leucothrix mucor* clade within the *Gammaproteobacteria*^28^, strongly suggested that filamentous bacteria could be chemoautotrophic sulfur oxidizers. The colonization of *T*. *benedii* by bacteria seems to be a regular phenomenon with seasonal variations: bacterial density is low during winter and spring and high during summer and fall when sulfide concentrations are highest^29^. Our results also showed a seasonal variability of *M*. *albidus* ectosymbionts, particularly filamentous bacteria, present exclusively in July 2016 and 2017 when sulfide concentration is higher due to algal decomposition. Numerous host species from different phyla are now known to harbour chemosynthetic symbionts and are present in a wide range of habitats from shallow-water coastal sediments to deep-sea, such as hydrothermal vents, whale falls, cold seeps, mud volcanoes and continental margins^1^. Most habitats in the deep-sea are dominated by chemosynthetic symbioses while at shallow-water vents and seeps this type of symbiosis could occur occasionally. Chemosynthetic symbioses have been found in shallow-water coastal sediments with high sulfide concentrations, such as in sea-grass beds^30^, mangrove muds^31,32^, sediments in upwelling regions^33^, and coastal mudflats of the North Atlantic^29^. More intriguingly, some low-sulfide habitats are colonized by a high diversity of chemosynthetic hosts like gutless oligochaetes, nematodes or bivalves, suggesting that a constant supply of sulfide might be more important than the absolute concentration itself^1^. The sampling collection site of this study is located within the old harbour of Roscoff, an unstable environment that experiences an extreme tidal range. Additionally, in summer, a high level of algal decomposition takes place at the sampling site, changing the chemical composition of the sediment at least in the first layer^34^ (<1 cm).

### Sulfur oxidation and sulfate reduction

In this study, we cloned a fragment of the alpha subunit of the adenosine-5-phosphosulfate reductase (AprA) gene, a key enzyme of the sulfur cycle, involved in both dissimilatory sulfate reduction and sulfur oxidation^35–38^, and generally used as a functional marker gene in phylogeny^39^. *AprA* sequences of *M*. *albidus* grouped within both SRB and SOB clades suggesting that the symbionts could use both reduction and oxidation pathways. A co-occurrence of SRB and SOB from an ectosymbiotic association has previously been described in the deep-sea, with the Yeti crab *Kiwa hirsuta*^40^ and the shrimp *Rimicaris exoculata*^41^, and in shallow water with the worm *T*. *benedii*^28^ and *Olavius algarvensis*^42^. Most *M*. *albidus* sequences and a *K*. *hirsuta* sequence were clustered in the SOB lineage I with *Gammaproteobacteria*, close to symbionts of *Bathymodiolus* sp (*Gammaproteobacteria*) and to *Thiocystis violacea*, also a *Gammaproteobacteria*. Then our results strongly suggest that the *AprA* SOB lineage I sequences from *M*. *albidus* belong to their *Gammaproteobacteria* ectosymbionts, and that this symbiont relies on sulfur oxidation as an energy source. Sulfate-reducing bacteria of *M*. *albidus* sequences were closely related to an *R*. *exoculata Deltaproteobacteria*-related lineage and *Desulfobulbus* spp., suggesting an affiliation to *Deltaproteobacteria*. The co-occurence seems to be a positive symbiosis with true benefit for the hosts in shallow water. Indeed, a motile nematode such as *M*. *albidus* probably migrates between sulfidic and oxygenated sediment layers, providing an alternance of conditions that allows both types of metabolism to occur.

### Potential role of the nematode-bacteria association

The filamentous bacteria observed in SEM and using FISH *Gammaproteobacteria* probe are most probably related to *Gammaproteobacteria* lineages retrieved as abundant using metabarcoding. They did not group with known chemosynthetic ectosymbionts of nematodes (Astomonematines and Stilbonematines) or oligochaete worms (*Olavius* and *Inanidrilus*) but instead with symbionts from deep-sea vent organisms such as shrimp, crabs, gastropods, and mussels. Metabarcoding results showed that many related *Campylobacterota* are members of the *Thiovulgaceae* family. *Thiovulgaceae* and *Thiotrichaceae* were generally identified as ectosymbionts harboured by hydrothermal vent hosts such as crustaceans^43^. A close relationship between ectosymbionts from deep-sea and shallow-water hosts is a rare occurrence, and this is the first example for the phylum Nematoda. These results are important as they suggest that host affiliation does not play a major role in ectosymbiotic association, unlike dynamic local environmental conditions. Only one other shallow-water study on the marine worm *T*. *benedii* had similar results, with filamentous bacteria affiliated to *Thiovulgaceae* and *Thiotrichaceae* related to deep-sea hydrothermal vent ectosymbionts. Unfortunately, the processes of colonization, acquisition or transmission of the symbionts are often still speculative, especially for ectosymbionts. In chemosynthetic symbioses, different types of transmission modes are possible: environmental (through a free-living population of symbiotic bacteria), horizontal (between organism sharing the same habitat) and vertical (passed down via the gametes^44^). Despite a lack of examples of ectosymbiotic associations for the smallest species (meiofauna), our results allow us to hypothesize that there is horizontal transmission. Indeed, ectosymbionts were observed on the cuticle of males and females, but juveniles were asymbiotic and we did not observe the same population of bacteria in the surrounding environment. In addition, nematodes moult and the epibionts would have to be re-acquired if there is no specific structure to host the bacteria before a moult.

The ectosymbiotic community of *M*. *albidus* has the potential for chemoautotrophic sulfur oxidation, although its role is unknown. Indeed, bacteria are used as a food source by nematodes but additional roles have also been hypothesized, such as a barrier against sulfide poisoning^19,45–48^ or nitrogen fixation, as in the coastal stilbonematid nematode *Laxus oneistus*^49^. Sulfur-oxidizing ectobacteria on marine nematodes, such as Stilbonematinae, cover the entire external body, suggesting a possible grazing activity by hosts on the symbionts^50^. *M*. *albidus* possesses a feeding structure with a full digestive system and its filamentous ectosymbionts are attached to its cuticle with only basal cells, thus providing just a small surface area for exchange. Therefore, it seems unlikely that ectosymbionts play an important role in the nutrition of this nematode, at least through cuticle transfer, as for other symbioses^51^. Another potential role is sulfide detoxification of the water surrounding the nematodes at microscale area. Such a function has been suggested for Stilbonematinae nematodes^19^ and for the freshwater cave amphipod *Niphargus ictus*^2^. For instance, in the nematode *Stilbonema majum*, aposymbiotic individuals died in 200 µM sulfide, whereas the same concentration did not affect the survival of symbiotic individuals^48^. High density for ectosymbiotic communities of *M*. *albidus* were only observed in July 2016 and 2017, suggesting an adaptation to a change in environmental conditions, such as an increase of sulfide concentration due to algae degradation in summer.

## Conclusions

Our results show that a shallow-water meiofaunal organism, *M*. *albidus*, has a specific microbial community, distinct from its surrounding environment and characterized by high seasonal variability. Ectosymbiotic associations with *Campylobacterota* have only been previously described in deep-sea invertebrates and one shallow-water oligochaete worm. Ectosymbiotic associations with *Gammaproteobacteria* are more widespread. However, the *M*. *albidus* gamma-ectosymbiont relative belongs to a deep-sea hydrothermal vent symbiotic bacterial clade, distantly related to other shallow-water nematode or worm-associated bacterial communities. The discovery of the *M*. *albidus* seasonal ectosymbiosis together with a gene involved in sulfur metabolism (*AprA*) suggests that environmental factors may play a crucial role in the biology and evolution of bacteria-meiofauna associations.

## Methods

### Study area and free-living marine nematode sorting

Anoxic sediment samples were collected manually at low tide in the old harbour of Roscoff (48°43′34.20′′N and 3°58′50.53′′W) on five visits (July 2016, November 2016, March 2017, April 2017 and July 2017). The top black layer of the sediment (<5 cm) was sieved in the field and quickly brought back to the laboratory where live nematodes were sorted under a stereomicroscope (M125; Leica, Wetzlar, Germany). We selected one of the most abundant species, *M*. *albidus*, identified based on its morphology. A set of specimens (males, females, and juveniles) was immediately frozen at −80 °C for later molecular analyses (both on the nematodes and their microbial diversity). Additional specimens were processed for SEM studies: these nematodes were fixed in glutaraldehyde 2.5% for 16 h at 4 °C^52^, then transferred to a sodium azide solution (0.065 g in 150 ml filtered sea water) and stored at 4 °C until use. Another set of nematodes was processed for FISH analyses: samples were fixed for 2 h in 3% formaldehyde (in sterile seawater) solution and rinsed with 1X phosphate-buffered saline (PBS)–sterile seawater solution (1:1). These samples were stored in absolute ethanol - 2X PBS solution (1:1) at −20 °C until use^53^. Additionally, in July 2017, triplicates of sediment and water were collected for metabarcoding of environmental bacteria.

All experiments performed in this study are summarized in Table 2.

**Table 2:** Summary of molecular experiments and microscopic observations used to characterize *Metoncholaimus albidus* and explore the bacterial diversity.

	*Metoncholaimus albidus*				Bacterial diversity					
	18S	28S	Cox1	AprA (clones)	*Ma* (NGS)	*Ma* (clones)	Sediment (NGS)	Water (NGS)	SEM	FISH
July 2016									5	4
November 2016	3	7	1		6				7	
March 2017	3	6			6	3 (228)				4
April 2017	2	6	2		6					
July 2017		7		4 (42)	7		3	3	6	5
N total samples	8	26	3	4	25	3	3	3	18	13

*Ma* = *Metoncholaimus albidus*; N = number; NGS = Illumina technology.

### Nematode morphological studies

To verify that all the nematodes belonged to the same species, we performed a detailed morphological investigation on a subset of the population (adults and juveniles). Several nematodes were mounted on slides for detailed morphological observation using the formalin–ethanol/glycerol technique^54,55^. Photos were captured on a Leica DM IRB microscope and a Zeiss AxioZoom microscope, each equipped with live camera (Image-Pro and Zen software, respectively).

### Free-living marine nematode DNA extraction, PCR and sequencing

Species assignment of the nematodes directly frozen at −80 °C was verified with a molecular approach. Total DNA was extracted from each nematode individually, using the Qiagen® DNeasy Blood & Tissue kit following the manufacturer’s instructions. Partial fragments of the nuclear 18S rRNA (597 bp), nuclear 28S rRNA gene (654 bp) and mitochondrial cytochrome c oxidase subunit 1 (cox1) (393 bp), were amplified with different primer sets (Table 3). Mitochondrial PCRs were performed on a Gene-Amp^TM^ PCR system 9700 thermocycler (Applied Biosystems, Forster City, CA, USA) in a final volume of 25 µl using the following mix: 2 µl extracted DNA was added to 5 µl 5X PCR buffer, 2.5 mM of each dNTP, 50 mM MgCl_2_, 15 µM and 0.1 µl Taq polymerase (5U/µl - Promega). Thermocycle profile included 5 min at 94 °C followed by 35 cycles of 30 s at 94 °C, 45 s at 54 °C and 1 min at 72 °C, followed by a final extension of 10 min at 72 °C. Nuclear 18S rRNA PCRs have the same protocol that the 28S rRNA already described by Bellec and colleagues^5^. All PCR products were run on a 0.8% agarose-TAE gel to verify the size of the amplicons. Purification and Sanger sequencing of PCR products were performed by Macrogen (South Korea). Chromatograms were ckecked and DNA sequences were assembled and edited using Geneious 8.1.9^56^ and all nucleotide differences were checked visually.

**Table 3:** Primers and Fluorescent probes used in this study.

Gene/Phylotype	Primer/Probe	Primer/Probe sequence (5′-3′)	References
18S rRNA	18S1.2a	CGATCAGATACCGCCCTAG	Bernard *et al*.^75^
18S rRNA	18Sr2b	TACAAAGGGCAGGGACGTAAT	
28S rRNA	D2Ab	ACAAGTACCGTGAGGGAAAGTTG	De Ley *et al*.^76^
28S rRNA	D3B	TCGGAAGGAACCAGCTACTA	
Cox1	JB3	TTTTTTGGGCATCCTGAGGTTTAT	Hu *et al*.^77^
Cox1	JB4.5	TAAAGAAAGAACATAATGAAATG	
AprA	APS1	TGGCAGATCATGATYMAYGG	Meyer and Kuever^39^
AprA	APS4	GCGCCAACYGGRCCRTA	
16S rRNA	E8F	AGAGTTTGATCATGGCTCAG	Lane *et al*.^78^
16S rRNA	U1492R	GTTACCTTGTTACGACTT	Cambon-Bonavita *et al*.^79^
*Eubacteria*	Eub338-I	GCTGCCTCCCGTAGGAGT	Amann *et al*.^80^
*Deltaproteobacteria*	Delta495a	AGTTAGCCGGTGCTTCCT	Loy *et al*.^81^
*Campylobacterota*	EPSY549	CAGTGATTCCGAGTAACG	Lin *et al*.^82^
*Gammaproteobacteria*	GAM42a	GCCTTCCCACATCGTTT	Manz *et al*.^83^
Non-sens	Non338	ACTCCTACGGGAGGCAGC	Wallner *et al*.^84^

### *AprA* gene amplification, cloning and sequencing

A fragment of the adenosine-5′-phosphosulfate reductase alpha subunit (AprA) (389 bp) was amplified (see Table 3 for primers). Amplifications were performed in a final volume of 25 µl using the following mix: 4 µl DNA was added to 5 µl 5X PCR buffer, 10 mM of each dNTP, 50 mM MgCl_2_, 20 µM of each primer and 0.1 µl Taq polymerase (5U/µl, Promega). The thermocycle profile included 4 min at 94 °C followed by 35 cycles of 1 min at 94 °C, 1 min at 58 °C and 1 min 72 °C, followed by a final elongation step of 10 min at 72 °C. All PCR products were purified using the NucleoSpin® Gel and PCR Clean-up (Macherey-Nagel, Düren, Germany) according to the manufacturer’s instructions. They were then cloned using the TOPO® TA cloning® kit (Invitrogen, Carlsbad, CA) following the manufacturer’s instructions. Sequences of positive clones were amplified by Eurofins Genomics (France).

### 16S rRNA bacterial diversity analyses through cloning after amplification

A partial fragment of the 16S rRNA bacterial gene (1484 bp) was amplified (see Table 3 for primers). Amplifications were performed in a final volume of 50 µl using the following mix: 4 µl DNA was added to 10 µl 5X PCR buffer, 10 mM of each dNTP, 100 mM MgCl_2_, 20 µM of each primer and 0.2 µl Taq polymerase (5U/µl - Promega). The thermocycle profile included 5 min at 94 °C followed by 30 cycles of 1 min at 94 °C, 1.5 min at 48 °C and 1 min 72 °C, followed by a final 6 min extension at 72 °C. All PCR products were checked on a 0.8% agarose–TAE gel to verify the size of the amplicons. Before cloning, all PCR products were purified using the NucleoSpin® Gel and PCR Clean-up kit (Macherey-Nagel, Düren, Germany) according to the manufacturer’s instructions. The purified fragments were the cloned using the TOPO® TA cloning® kit (Invitrogen, Carlsbad, CA) following the manufacturer’s instructions. Positive clones were prepared for sequencing by Eurofins Genomics (France).

### 16S rRNA bacterial diversity analyses by Next Generation Sequencing

DNA from 25 *M*. *albidus* (based on morphological and genetic identification) of four samples (November 2016, March 2017, April 2017 and July 2017) were sent to MR DNA (Shallowater, TX, USA) for amplification of prokaryotic diversity based on 16S rRNA gene. DNA from sediment (July 2017) and water (July 2017) were also used as environmental references. Total DNA was extracted from sediment using the Qiagen® DNeasy PowerMax Soil kit and from water using the Qiagen® DNeasy PowerWater kit following the manufacturer’s instructions. Three negative controls (blank sample from each extraction: nematode, sediment, water) were also used for sequencing.

Sequencing (a 450 bp fragment of the 16S rRNA gene) was performed on the Illumina MiSeq platform^57^ using 2 × 300 bp chemistry (for details see Bellec *et al*.^5^). We selected a variable region of the 16S rRNA (V3-V4) frequently used for analyses the microbial diversity^58^.

### Bioinformatics analyses

Prokaryotic 16S rRNA paired-end reads were merged using USEARCH^59^ after q25 trimming of both ends. The resulting 16S reads were processed using the Find Rapidly OTU with the Galaxy Solution (FROGS) v2 pipeline^60^. In short, the barcode was removed, and reads <380 bp as well as containing ambiguous sites were removed. Next, reads were clustered into *de novo* operational taxonomic units (OTUs) using Swarm^61^, with an aggregation distance of 3. Chimera were then removed with VSEARCH^62^. Additionally, filters were applied to the OTUs: one for abundance, with an optimal threshold of 0.005%^63^, and one for OTU occurrence (sequences had to be present at least in three samples). The OTUs finally selected were taxonomically assigned by BLASTn+^64^ using the Silva release 128 reference database^65^. Finally, filtrations on BLAST taxonomic affiliation were performed, with a minimum coverage of 95% and a minimum identity of 60%. OTU structure, visualization, composition analysis and alpha diversity indices were performed using phyloseq^66^ available through FROGS. Parts of the visualization were also done with through Phinch framework^67^. Venn diagrams were produced using the Venny v2.1 software (http://bioinfogp.cnb.csic.es/tools/venny/index.html).

### Scanning electron microscopy observations

Eighteen *M*. *albidus* were used for SEM observations to confirm the presence of prokaryotes attached on the cuticle of the nematodes (Table 2). After fixation (see *Nematode sorting* section), nematodes were postfixed in 0.8% osmium tetroxide 20 h at 4 °C and then dehydrated through an ethanol series. Nematodes were desiccated with a critical-point dryer (CPD 300; Leica, Wetzlar, Germany) and then mounted on a specimen stub. They were gold-coated using an SCD 040 (Blazers Union, Blazers, Liechtenstein). Observations were made with a Quanta 200 MK2 microscope (FEI, Hillsboro, OR, USA) and the xT microscope software (FEI). Scanning electron micrographs were used for morphological identification.

### Fluorescence *in situ* hybridizations

FISH was performed to confirm the occurrence of prokaryotes on 13 nematodes (Table 2). In the field or quickly after sampling, some nematodes were fixed (see *Nematode sorting* section). Later, in the laboratory, they were hybridized with universal probes (Eurogentec, Liège, Belgium) (Table 3). Nematodes were rinsed in a 30% formamide buffer^53^ and incubated in a final volume of 30 µl hybridization buffer containing 30% formamide and 3 µl of each probe (8 µM) for 3.5 h at 46 °C. After that, the nematodes were rinsed in a washing buffer for 45 min at 48 °C. This step was ended by a final wash in milliQ water at room temperature for 10 min. After a quick drying period, the labelled organisms were mounted on a slide in an anti-fade mounting medium (SlowFade® Gold anti-fade reagent, Invitrogen) containing DAPI (4′6-diamidino-2-phenylindole, dilactate), a DNA intercalary agent. Observations were performed using the Imager.Z2 microscope (Zeiss, Oberkochen, Germany) equipped with an Apo-Tome slider module (Zeiss) and Colibri light technology (Zeiss) and using an AxioCam MRm (Zeiss) camera. Micrographs were analysed using the Zen (Zeiss) software.

### Phylogenetic reconstructions

#### Gammaproteobacterial phylogeny

Two *Gammaproteobacteria*-related sequences of *M*. *albidus* from July 2017, 46 *Gammaproteobacteria* from GenBank, and three outgroups (*Campylobacterota*) were used in the analysis. The dataset of the 16S rRNA gene was aligned with MUSCLE as implemented in Geneious 8.1.9^54^ and then processed in Gblocks© (version 0.91b) to remove gaps (425 bp final). Phylogenetic reconstructions were performed with two methods: Bayesian inference (BI) using Mr Bayes 3.2.6^68^, and Maximum likelihood (ML) using RAxML BlackBox^69^ on the CIPRES Science Gateway^70^. The best-fitting model of evolution was computed with jmodeltest v.2.1.6^71^. Bayesian analysis was carried out with four chains of 5 × 10^6^ generations, trees sampled every 500 generations, a GTR + I + G model and burn-in value set to 25% of the sampled trees. We checked that standard deviation of the split frequencies fell below 0.01 and confirmed convergence of the runs to ensure convergence in the tree search using Tracer v1.6 (http://tree.bio.ed.ac.uk/software/tracer/). The tree was visualized with FigTree v1.4.3 (http://tree.bio.ed.ac.uk/software/figtree/).

#### *AprA* phylogeny

Four representative sequences of AprA from July 2017, 40 AprA sequences from GenBank and three outgroups (*Archaeoglobus*) were used in this study. A dataset of amino acid sequences was aligned and processed in Gblocks© (125 aa final). The best-fitting model of evolution was computed with SMS implemented in PhyML^72^. A maximum likelihood reconstruction was performed using PhyML 3^73^ with a LG + G model and 1000 bootstraps. BI was performed as described above with four chains of 5 × 10^6^ generations, trees sampled every 500 generations, an aa mixed model and burn-in value set to 25% of the sampled trees.

#### M. albidus phylogeny

To determine the phylogenetic position of the nematodes, we used eight representative sequences, 39 published *Oncholaiminae* and three outgroups (*Bathyeurystomina*). A concatenated alignment of partial 18S, 28S and cox1 was performed with MUSCLE using Geneious for a final length of 1607 bp (596, 612 and 399 for 18S, 28S and cox1, respectively). Partition schemes and evolutionary models were selected via the Bayesian Information Criterion calculated in PARTITION FINDER v1.1.1^74^: for 18S the K80 + G model; 28S and cox1 1st codon partitions the GTR + G model; for cox1 2nd codon partition the F81 model and for cox1 3rd codon partition the HKY + G model. BI was performed with four chains of 5 × 10^6^ generations, trees sampled every 100 generations and a burn-in value set at 25% of the sampled trees. BI and ML (with RAxML) were obtained using the same procedures as described above.

#### Abbreviations

AprA: adenosine-5′-phosphosulfate reductase alpha subunit; BI: Bayesian inference; Cox I: cytochrome c oxidase subunit; DAPI: 4′6-diamidino-2-phenylindole; DNA: Deoxyribonucleic acid; FISH: Fluorescence *In Situ* Hybridization; MANOVA: multivariate analysis of variance; ML: Maximum likelihood; NGS: Next-generation sequencing; OTU: Operational Taxonomic Unit; PBS: phosphate-buffered saline; RNA: Ribonucleic acid; SEM: Scanning Electron Microscopy. SOB: sulfur-oxidizing bacteria; SRB: sulfate-reducing bacteria.

## Supplementary information

Supplementary file

## Data Availability

The data supporting the results of this article are available in the NCBI SRA repository (Accession SRP152813, BioProject PRJNA480222). All sequences are available in GenBank under accession numbers MH587708 – MH587741 (18S and 28S); MH588057 – MH588058 (16S Gammaproteobacteria); MH59346 – MH593852 (Cox1 and AprA).
