# Supplementary material for: Chemosynthetic ectosymbionts associated with a shallow-water marine nematode
Source: Sci Rep. 2019 May 7;9:7019. doi: 10.1038/s41598-019-43517-8 (PMC6505526; doi:10.1038/s41598-019-43517-8)
Supplement: Supplementary file 1 — Supplementary file [file 41598_2019_43517_MOESM1_ESM.pdf]

Chemosynthetic ectosymbionts associated with a shallow-water marine nematode

Laure Bellec[1,2,3,4], Marie-Anne Cambon Bonavita[2,3,4], Stéphane Hourdez[5,6], Mohamed Jebbar[3,4], Aurélie Tasiemski[7], Lucile Durand[2,3,4], Nicolas Gayet[1], Daniela Zeppilli[1]✉

[1] *IFREMER, Centre Brest, REM/EEP/LEP, ZI de la pointe du diable, CS10070, 29280 Plouzané, France*

[2] *IFREMER, Univ Brest, CNRS, Laboratoire de Microbiologie des Environnements Extrêmes, F-29280, Plouzané, France*

[3] *CNRS, UMR 6197-Laboratoire de Microbiologie des Environnements Extrêmes (LM2E), Institut Universitaire Européen de la Mer (IUEM), Technopole Brest-Iroise, Plouzané, France*

[4] *Université Bretagne Occidentale (UBO), UMR 6197 - Laboratoire de Microbiologie des Environnements Extrêmes (LM2E), Institut Universitaire Européen de la Mer (IUEM), Technopole Brest-Iroise, Plouzané, France*

[5] *Station biologique de Roscoff, UMR 7144 CNRS-SU, Adaptation and Biology of Invertebrates in Extreme Environment team, Place G. Teissier, 29680 Roscoff, France*

[6] *Present address : Observatoire Oceanologique de Banyuls-sur-Mer, UMR 8222 CNRS-SU, 1 avenue Pierre Fabre, 66650 Banyuls-sur-Mer, France*

[7] *Université Lille, CNRS, UMR 8198 - Evo-Eco-Paleo, SPICI group, 59000 Lille, France*

✉ Corresponding author:

Telephone: +33(0)298224382;

Fax: +33(0)298224757;

e-mail: Daniela.Zeppilli@ifremer.fr

Supplementary Information

Supplementary file 1. Morphology and molecular identity of the free-living marine nematode.

Metoncholaimus is a free-living marine nematode with over 21 species described to date (WoRMS, http://www.marinespecies.org/). This genus belongs to the most diverse subfamily, Oncholaiminae, which comprises 11 genera. The species *M. albidus* is characterized by 99 long spicules, the presence of a gubernaculum, and a well-developed demanian system with single uvette and double monoliform terminal. The *M. albidus* specimens sampled in Roscoff had the following characteristics: females were longer and wider than males (6219 vs 5293 µm in max length and 68 vs 55 µm in max width); males measured between 4635 and 5293 µm in length (Supplementary file 2); cuticle presented very fine striation; cervical setae were present up from the nerve ring towards the anterior end; the six conical lips were deeply separated, each bearing a minute rounded inner labial papilla; ten setae (six outer labial and four cephalic) in one circle; an wide and deep buccal cavity characterized by sclerotized walls; three unequal teeth (onchs): two small teeth (dorsal and right ventral sublateral) and one large left ventrosublateral tooth; cup-shaped/pocket-like amphideal fovea; vulva at 66–70% of the body length; very long spicules (386–411 µm); presence of a Demanian system in females with the presence of an external girdle (Supplementary file 2). The Demanian organ has a secretory function and can become a girdle fixed around the pre-anal narrowing of the female body. Functions hypothesized for the girdle include it being a sexual attractant and protection for deposited eggs. SEM micrographs showed that in *M. albidus* the girdle is closed (Supplementary file 2A-F), attached to the body (Supplementary file 2F) and characterized by two lines of regular pores (Supplementary file 2B).

Measurements of *M. albidus* (in µm). Abbreviations are as follows: a (de Man index), body length/maximum body diameter; a.b.d., anal body diameter; b (de Man index), body

length/oesophagus length; c (de Man index), body length/tail length; c.b.d, corresponding body diameter; V, vulva distance from anterior end of body; V%, V/total body length.

Characters	Males min-max	Females min-max	Juveniles min-max
**Total body lenght**	4635-5293	2572-6219	1942-4914
**Head diameter**	31-35	29-37	22-34
**Length of subcephalic setae**	6,4-9,5	6,3-7,7	5,3-9,6
**Nerve ring from anterior end**	564-645	546-714	557-593
**Nerve ring c.b.d.**	32-41	42-60	28-42
**Excretory pore from anterior**	86-109	99,1	89-109
**Oesophagus length**	540-614	518-641	496-537
**Oesophagus c.b.d.**	32-55	50-68	35-67
**Maximum body diameter**	85-99	74-1301	51-84
**Spicule length**	386-411	/	/
**Anal diameter**	57-66	47-90	32-87
**Tail length**	289-336	226-306	177-354
**Tail length**/a.b.d.	4,4-5,8	3,4-4,8	7,3-11
**Vulva from anterior**	/	1692-4179	/
**Vulva c.b.d.**	/	89,1-148	/
V%	/	66-70	/
a	47-62	30-65	38-71
b	7,6-9,3	5,0-10,3	3,9-9,2
c	15-18	11-24	11-18

Supplementary file 2. Scanning electron micrographs centred on the girdles of three *M. albidus* females. A) general view of the posterior region including the tail, B) detail on the girdle porous, C-F) different views of girdles covered by bacteria.

Supplementary file 3. Phylogenetic tree of Oncholaiminae performed with three partial genes (18S rRNA, 28S rRNA and cox1) by BI and ML. For simplicity, only the BI tree is shown; the ML tree has the same topology. The numbers are posterior probabilities (BI) and

bootstrap proportions (ML) reflecting clade support (value below 75 are indicated by dashes). Specimens from this study are shown in bold. Three *Bathyeurystomina* were used as outgroup. Accordingly, the nematodes from the old harbour of Roscoff formed a clade the maximum support thus representing a single species. *M. albidus* clade grouped with four sequences representing *Oncholaimus* sp. and the only sequence representing the genus *Metoncholaimus* available in GenBank to date. A sister group was formed by all *Viscosia* and most *Oncholaimus*.

Supplementary file 4. Cloning versus metabarcoding

Summary of 16S rRNA diversity of three *M. albidus* with two methods (NGS Illumina and Cloning).

	**NGS**			**Cloning**		
	**Ma_Rm03**	**Ma_Rm32**	**Ma_Rm34**	**Ma_Rm03**	**Ma_Rm32**	**Ma_Rm34**
No. Reads/Clones	337,595	109,473	85,353	67	87	74
No. OTUs	165	148	133	8	19	22
Coverage (%)*				100	97.7	95.9
Relative frequency of bacteria (%)						
Alphaproteobacteria	<1	1.7	<1		1	
Betaproteobacteria	1.7	<1	<1			3
Deltaproteobacteria	<1	<1	<1		1	
Epsilonbacteraeota	<1	28.2	13.5		5	4
Gammaproteobacteria	25.8	50.1	60.7	12	36	73
Actinobacteria	<1	<1	8.7			1
Bacteroidetes	14.8	5.1	9.2	39	5	1
Cyanobacteria	1	<1	1.1			
Firmicutes	<1	<1	4.3			3
Gracilibacteria	<1	<1	<1	4		
Lentisphaerae	3.9	9.2	<1	36	53	14
Spirochaetae	38.8	<1	<1	9		1

*Calculated according to the formula: coverage = [1-(n1xN)]x 100 with n1 = number of OTUs consisting of only one sequence and N = number of all clones. Clones were assigned to the same OTU if they shared 97% 16S rRNA sequence similarity with each other.

Results showed that sequences produced by cloning or metabarcoding were related to the same lineages for the three *M. albidus*, however with differences on their relative abundance. For example, *Campylobacterota* was highly detected by metabarcoding compared to cloning for nematodes Ma_Rm32 and Ma_Rm34. Conversely, *Lentisphaerae* was highly recovered by

cloning but not by metabarcoding. Such differences could be due to the stringent filters used during the bioinformatic procedures. Metabarcoding allowed the detection of more lineages, especially those with relative low abundance, such as *Actinobacteria*, *Firmicutes*, and *Cyanobacteria*. The fact that cloning failed to detect some proteobacterial lineages could be due to PCR bias. Furthermore, cloning is not a quantitative method.

Supplementary file 5. Venn diagrams between blank and each environment (nematode, sediment and water). Only significant OTU (>1% relative abundance of total reads) were used.

Supplementary file 6. Number of reads (Nb) at different stages of processing.

Samples ID	Nb after quality filter	Final Nb (after affiliation)
Water1	166,469	101,145
Water2	181,827	114,791
Water3	164,092	84,367
Ma_Ra12	240,646	216,059
Ma_Ra2	93,388	82,371
Ma_Ra3	177,896	159,873
Ma_Ra4	130,918	114,548
Ma_Ra5	303,668	262,719
Ma_Ra9	187,993	165,491
Ma_Rj11	226,598	196,981
Ma_Rj12	368,36	318,243
Ma_Rj13	225,2	195,99
Ma_Rj14	256,079	216,591
Ma_Rj15	231,975	199,36
Ma_Rj16	226,78	191,492
Ma_Rj17	117,222	98,921
Ma_Rm03	380,267	337,595
Ma_Rm04	43,884	39,593
Ma_Rm31	165,676	142,931
Ma_Rm32	125,449	109,473
Ma_Rm34	99,333	85,353
Ma_Rm36	187,448	160,735
Ma_Ro10	166,961	107,118
Ma_Ro54	133,666	115,912
Ma_Ro55	106,947	93,038

Ma_Ro58	78,885	68,059
Ma_Ro59	197,146	168,865
Ma_Ro9	138,666	122,395
Sediment1	159,657	81,382
Sediment2	114,208	45,052
Sediment3	135,232	52,991
**Total**	**5532,158**	**4449,434**

Supplementary file 7. Taxonomies.

Samples ID	Nb phylum	Nb class	Nb order	Nb family	Nb genus	Nb species
Water1	17	33	67	108	213	267
Water2	18	34	67	105	209	262
Water3	17	35	74	120	229	286
Ma_Ra12	12	23	48	81	130	152
Ma_Ra2	13	24	43	69	92	106
Ma_Ra3	11	20	40	65	95	108
Ma_Ra4	14	24	46	80	128	150
Ma_Ra5	15	27	54	93	157	188
Ma_Ra9	14	25	47	86	125	143
Ma_Rj11	17	33	65	96	158	173
Ma_Rj12	15	28	59	96	169	198
Ma_Rj13	14	30	59	99	159	183
Ma_Rj14	16	31	62	97	172	201
Ma_Rj15	12	25	59	93	159	184
Ma_Rj16	12	25	54	88	155	173
Ma_Rj17	21	40	73	110	202	235
Ma_Rm03	14	22	48	81	133	165
Ma_Rm04	12	17	33	49	73	89
Ma_Rm31	13	22	47	79	132	161
Ma_Rm32	12	21	41	69	122	148
Ma_Rm34	11	20	41	69	114	133
Ma_Rm36	15	27	54	83	144	174
Ma_Ro10	22	39	71	108	193	225
Ma_Ro54	15	27	52	79	125	131
Ma_Ro55	14	25	47	65	101	109
Ma_Ro58	11	20	45	64	104	117
Ma_Ro59	15	30	63	91	150	170
Ma_Ro9	14	23	46	64	109	122
Sediment1	21	42	75	116	211	259
Sediment2	21	40	73	110	207	249
Sediment3	19	38	71	111	208	250

Supplementary file 8. Diversity of summer samples at Roscoff. A) Rarefaction curves for 7 *M. albidus*, 3 water and 3 sediment samples, B) Venn diagram with significant OTU (> 0.1% of total sequences) for the 3 environments.

Supplementary file 9. Fraction of total reads and their taxonomic assignment in 25 *M. albidus* for *Deltaproteobacteria*, *Campylobacterota* and *Gammaproteobacteria*. A) Affiliation at the genus level for *Gammaproteobacteria*, B) Affiliation at the genus level for *Campylobacterota*, C) Affiliation at the genus level for *Deltaproteobacteria* n = numbers of OTU.

Supplementary file 10. FISH of bacteria from *M. albidus* (July 2017), breakdown of a co-hybridization cliché. A) In blue, DAPI-stained filamentous bacteria and host nuclei (on the top left corner of each picture); B) in yellow, filamentous bacteria hybridized with the general probe targeting Eubacteria; C) in green, filamentous bacteria hybridized with the specific probe targeting *Gammaproteobacteria;* D) merge picture of the three previously cited channels showing turquoise filamentous bacteria co-hybridized with Eubacteria and *Gammaproteobacteria* probes together with DAPI staining.

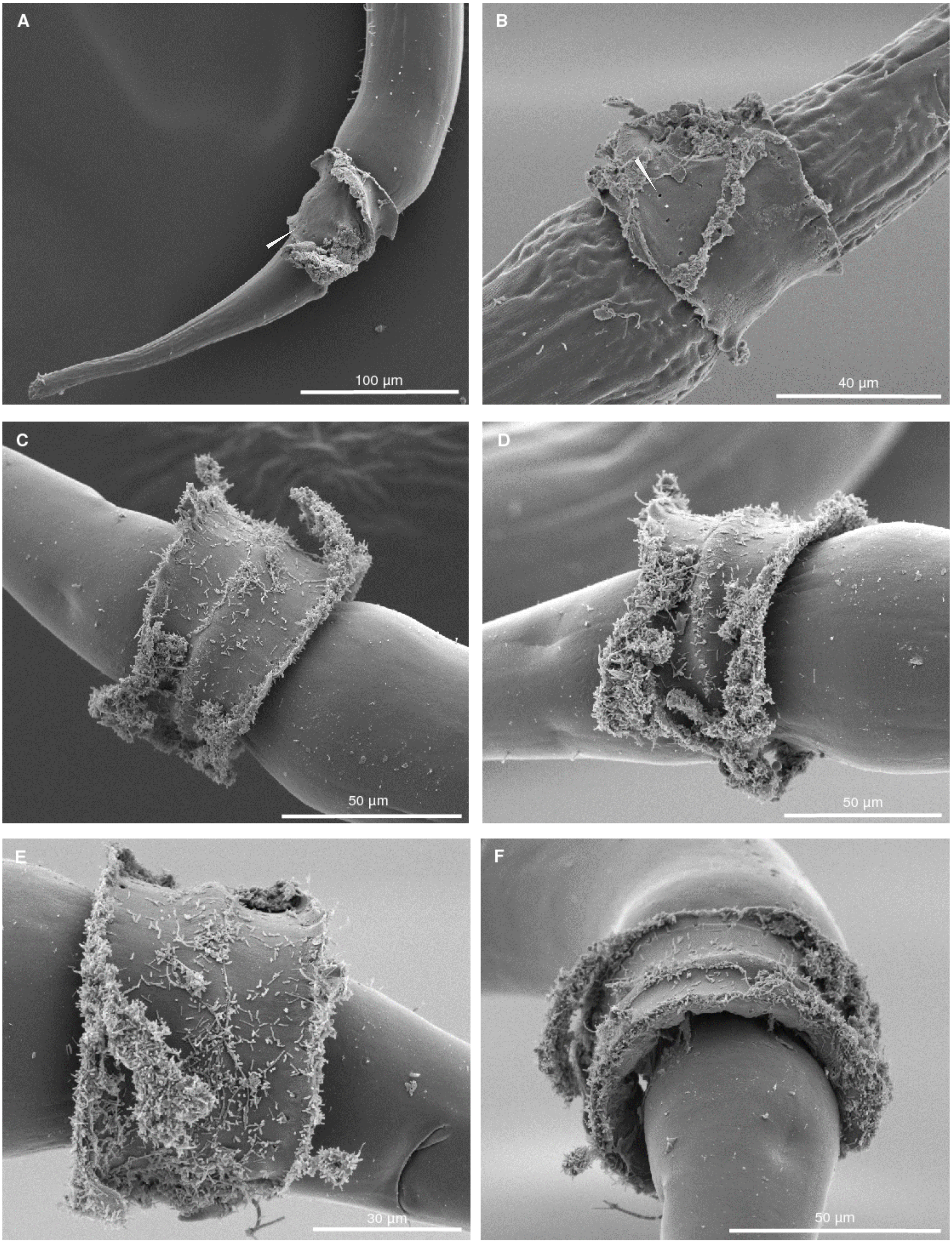
A
100 µm
B
40 µm
C
50 µm
D
50 µm
E
30 µm
F
50 µm

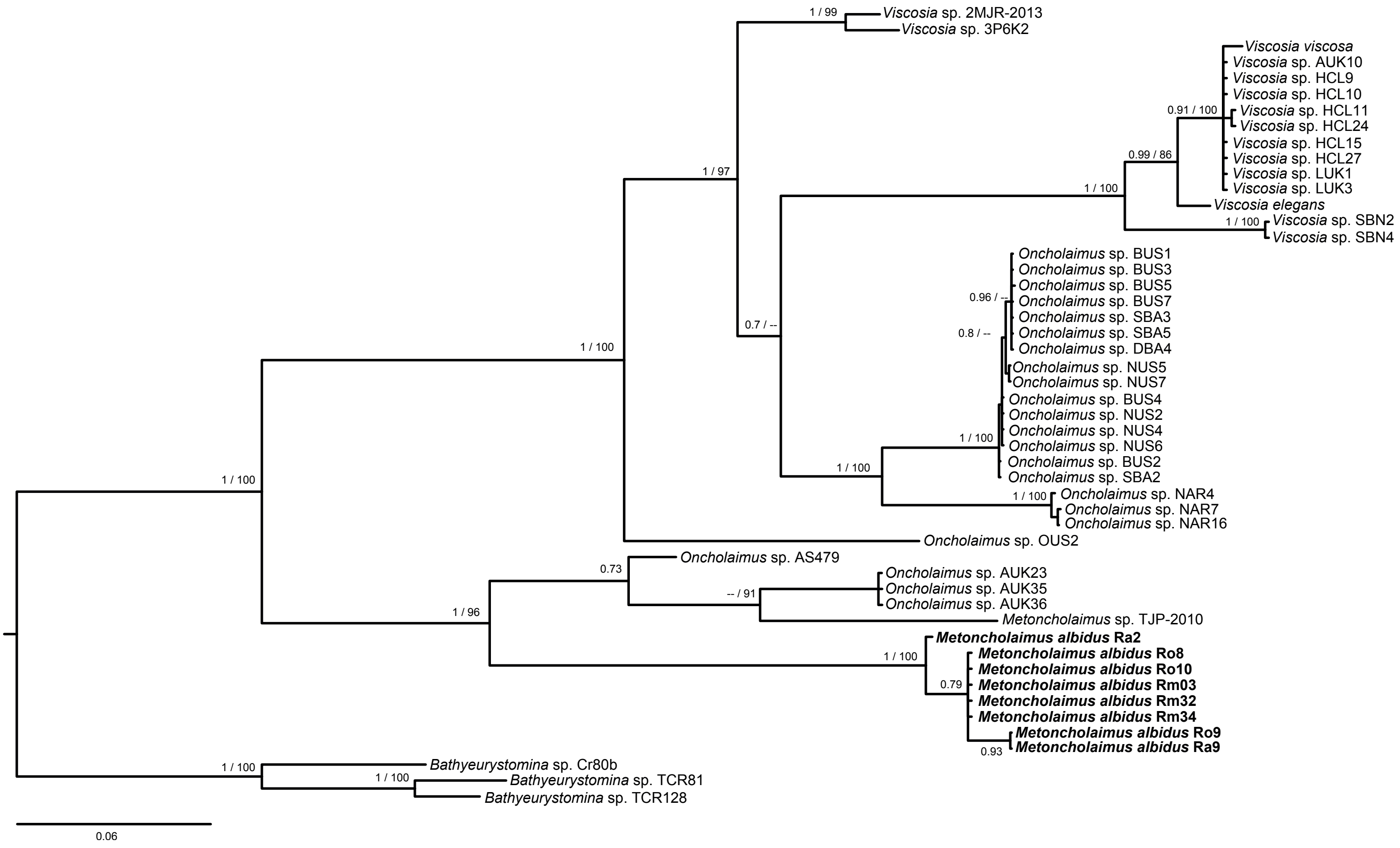

Viscosia sp. 2MJR-2013
Viscosia sp. 3P6K2
Viscosia viscosa
Viscosia sp. AUK10
Viscosia sp. HCL9
Viscosia sp. HCL10
Viscosia sp. HCL11
Viscosia sp. HCL24
Viscosia sp. HCL15
Viscosia sp. HCL27
Viscosia sp. LUK1
Viscosia sp. LUK3
Viscosia elegans
Viscosia sp. SBN2
Viscosia sp. SBN4
Oncholaimus sp. BUS1
Oncholaimus sp. BUS3
Oncholaimus sp. BUS5
Oncholaimus sp. BUS7
Oncholaimus sp. SBA3
Oncholaimus sp. SBA5
Oncholaimus sp. DBA4
Oncholaimus sp. NUS5
Oncholaimus sp. NUS7
Oncholaimus sp. BUS4
Oncholaimus sp. NUS2
Oncholaimus sp. NUS4
Oncholaimus sp. NUS6
Oncholaimus sp. BUS2
Oncholaimus sp. SBA2
Oncholaimus sp. NAR4
Oncholaimus sp. NAR7
Oncholaimus sp. NAR16
Oncholaimus sp. OUS2
Oncholaimus sp. AS479
Oncholaimus sp. AUK23
Oncholaimus sp. AUK35
Oncholaimus sp. AUK36
Metoncholaimus sp. TJP-2010
Metoncholaimus albidus Ra2
Metoncholaimus albidus Ro8
Metoncholaimus albidus Ro10
Metoncholaimus albidus Rm03
Metoncholaimus albidus Rm32
Metoncholaimus albidus Rm34
Metoncholaimus albidus Ro9
Metoncholaimus albidus Ra9
Bathyeurystomina sp. Cr80b
Bathyeurystomina sp. TCR81
Bathyeurystomina sp. TCR128
1 / 99
0.91 / 100
0.99 / 86
1 / 100
1 / 97
0.96 / --
0.8 / --
0.7 / --
1 / 100
0.73
-- / 91
1 / 96
0.79
0.93
1 / 100
0.06

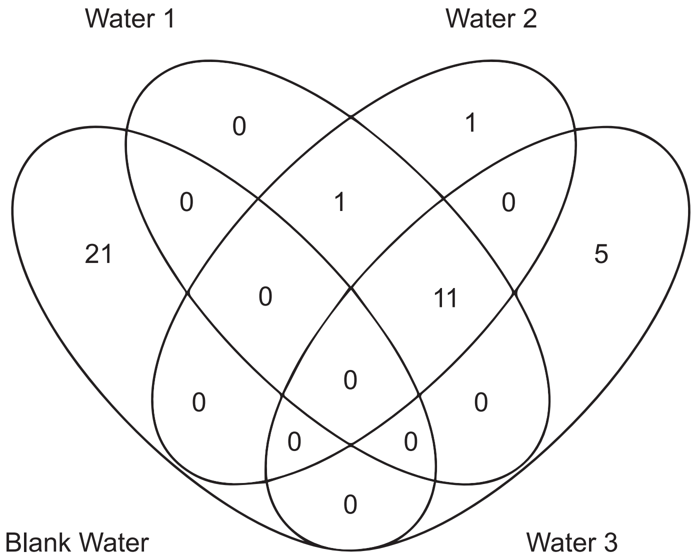
Water 1
Water 2
Blank Water
Water 3
21
0
1
5
0
1
0
0
11
0
0
0
0
0
0

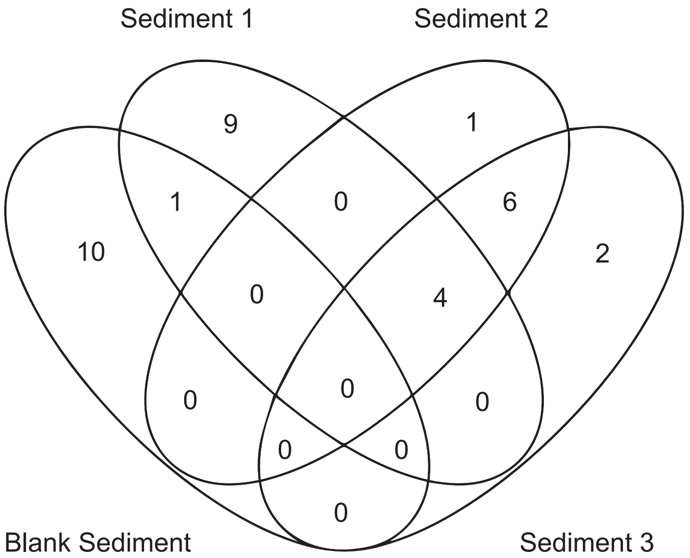
Sediment 1
Sediment 2
Blank Sediment
Sediment 3
10
9
1
2
1
0
6
0
4
0
0
0
0
0
0

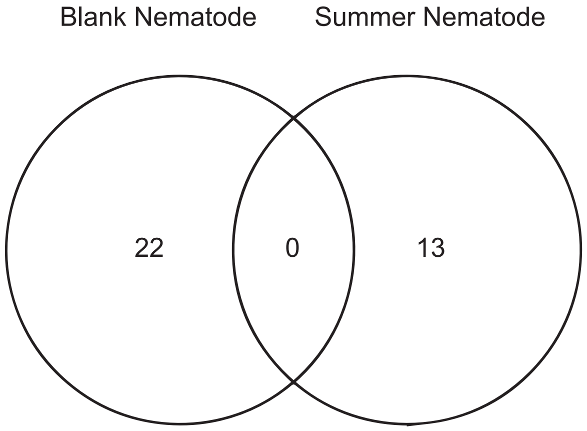
Blank Nematode
Summer Nematode
22
0
13

A

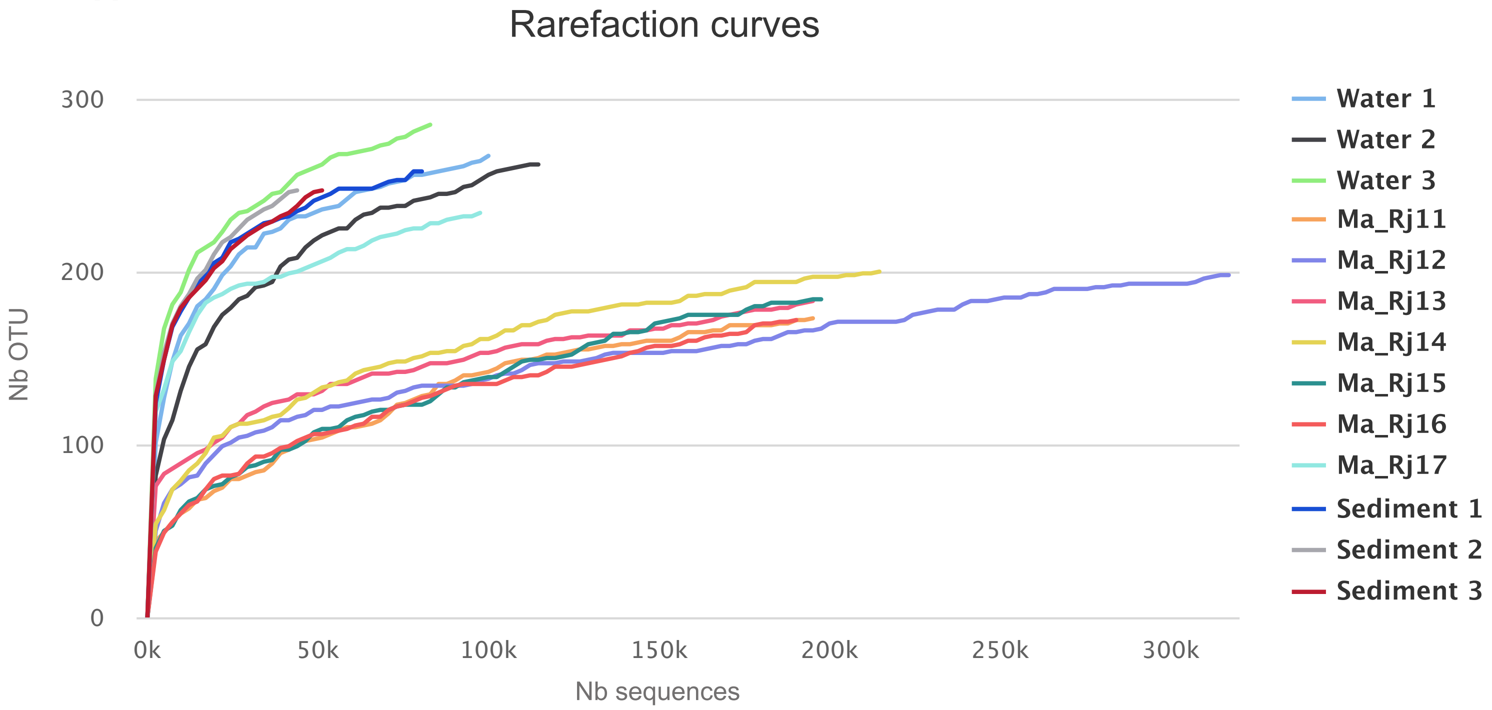

Rarefaction curves
Nb OTU
Nb sequences
0
100
200
300
0k
50k
100k
150k
200k
250k
300k
Water 1
Water 2
Water 3
Ma_Rj11
Ma_Rj12
Ma_Rj13
Ma_Rj14
Ma_Rj15
Ma_Rj16
Ma_Rj17
Sediment 1
Sediment 2
Sediment 3

B

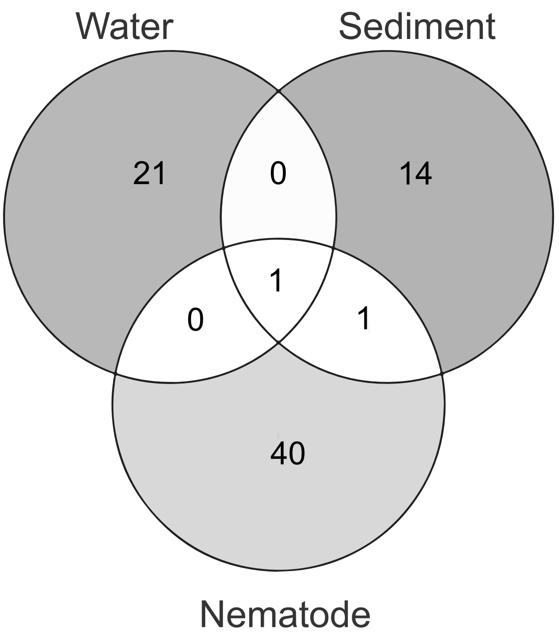

Water
Sediment
Nematode
21
0
14
1
0
1
40

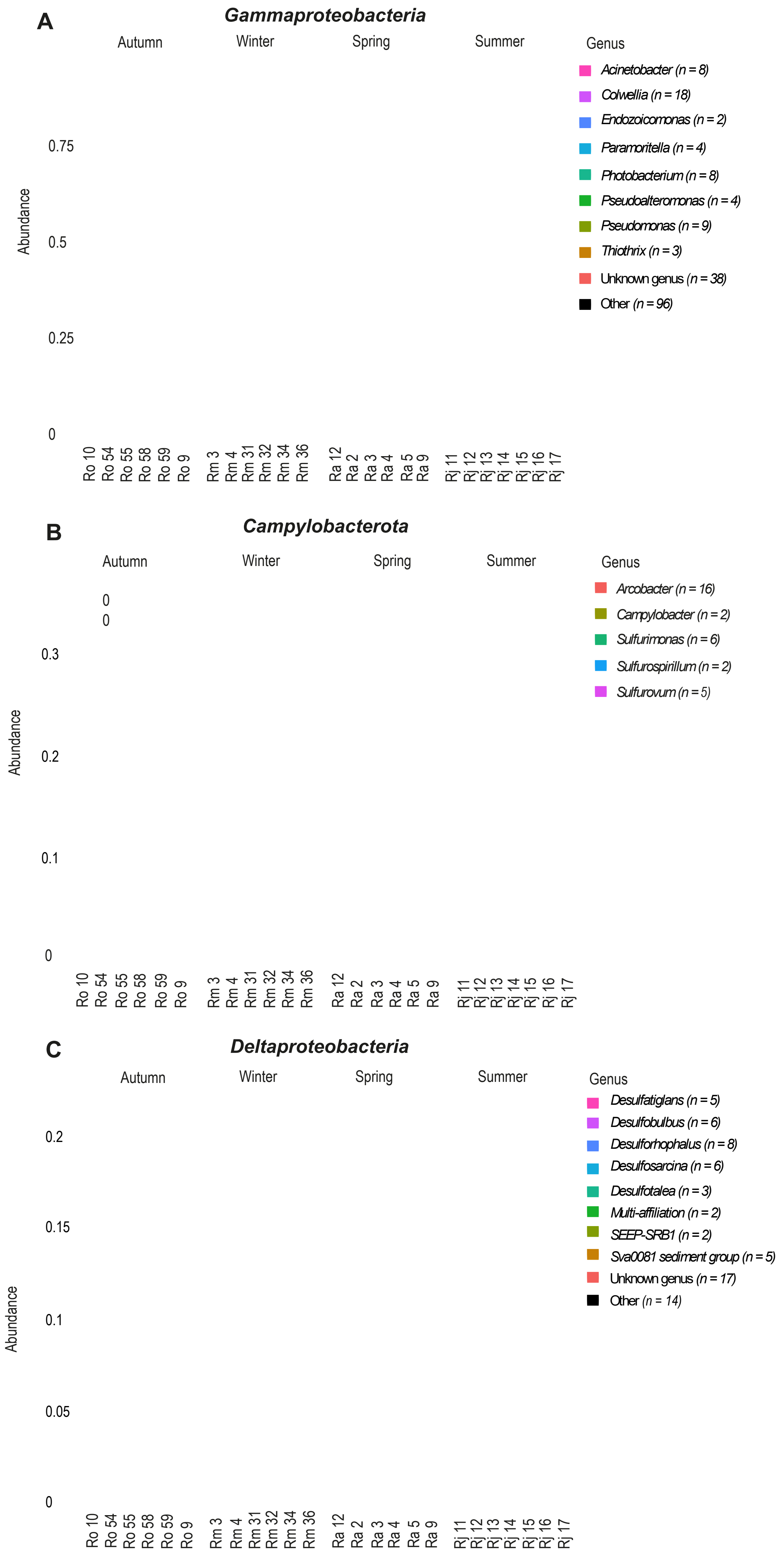
A
Gammaproteobacteria
Autumn
Winter
Spring
Summer
Abundance
0.75
0.5
0.25
0
Ro 10
Ro 54
Ro 55
Ro 58
Ro 59
Ro 9
Rm 3
Rm 4
Rm 31
Rm 32
Rm 34
Rm 36
Ra 12
Ra 2
Ra 3
Ra 4
Ra 5
Ra 9
Rj 11
Rj 12
Rj 13
Rj 14
Rj 15
Rj 16
Rj 17
Genus
Acinetobacter (n = 8)
Colwellia (n = 18)
Endozoicomonas (n = 2)
Paramoritella (n = 4)
Photobacterium (n = 8)
Pseudoalteromonas (n = 4)
Pseudomonas (n = 9)
Thiothrix (n = 3)
Unknown genus (n = 38)
Other (n = 96)
B
Campylobacterota
Autumn
Winter
Spring
Summer
0
0
Abundance
0.3
0.2
0.1
0
Ro 10
Ro 54
Ro 55
Ro 58
Ro 59
Ro 9
Rm 3
Rm 4
Rm 31
Rm 32
Rm 34
Rm 36
Ra 12
Ra 2
Ra 3
Ra 4
Ra 5
Ra 9
Rj 11
Rj 12
Rj 13
Rj 14
Rj 15
Rj 16
Rj 17
Genus
Arcobacter (n = 16)
Campylobacter (n = 2)
Sulfurimonas (n = 6)
Sulfurospirillum (n = 2)
Sulfurovum (n = 5)
C
Deltaproteobacteria
Autumn
Winter
Spring
Summer
Abundance
0.2
0.15
0.1
0.05
0
Ro 10
Ro 54
Ro 55
Ro 58
Ro 59
Ro 9
Rm 3
Rm 4
Rm 31
Rm 32
Rm 34
Rm 36
Ra 12
Ra 2
Ra 3
Ra 4
Ra 5
Ra 9
Rj 11
Rj 12
Rj 13
Rj 14
Rj 15
Rj 16
Rj 17
Genus
Desulfatiglans (n = 5)
Desulfobulbus (n = 6)
Desulforhophalus (n = 8)
Desulfosarcina (n = 6)
Desulfotalea (n = 3)
Multi-affiliation (n = 2)
SEEP-SRB1 (n = 2)
Sva0081 sediment group (n = 5)
Unknown genus (n = 17)
Other (n = 14)

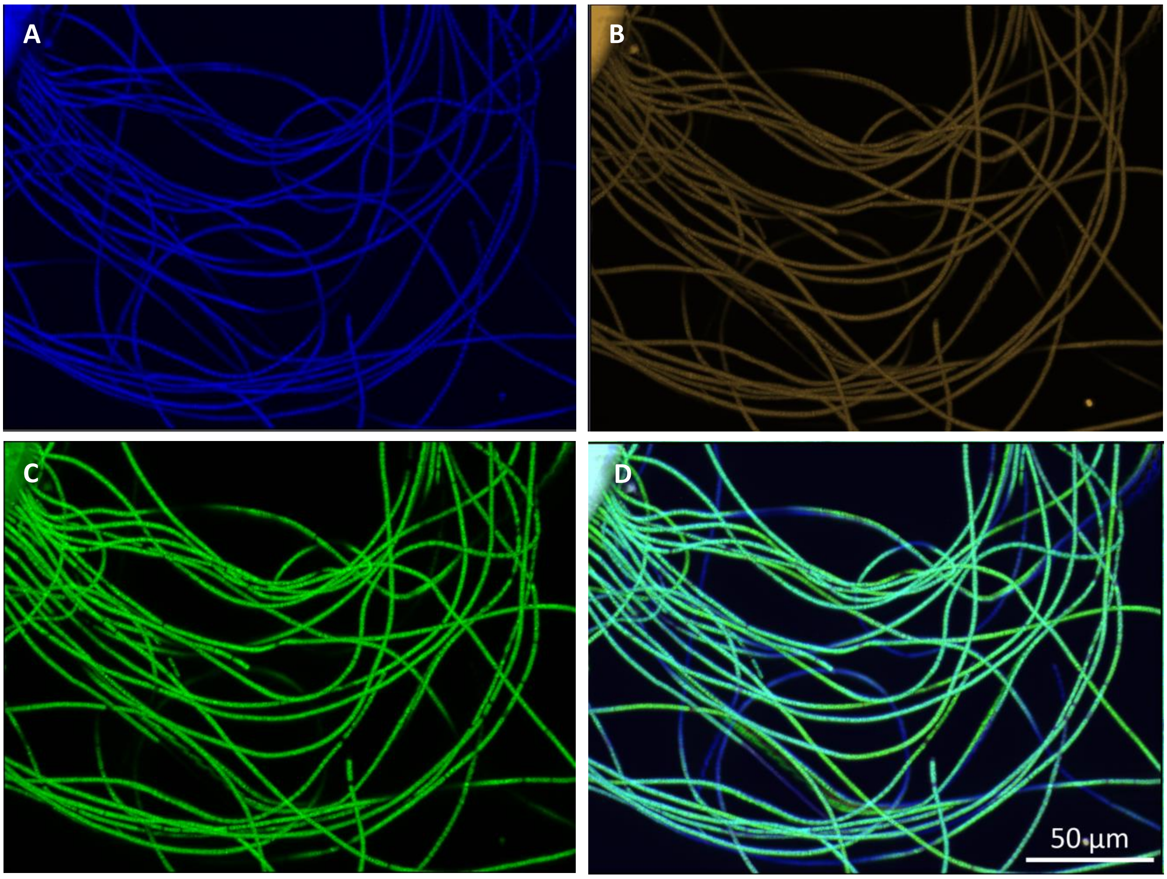
A
B
C
D
50 µm
